# Nanoscale Tracking of the High-Temperature Spin-State Transition in LaCoO_3_

**DOI:** 10.1021/acs.nanolett.5c03863

**Published:** 2025-11-05

**Authors:** Michelle A. Smeaton, Elena Salagre, Elliot J. Fuller, Lance M. Wheeler, Katherine L. Jungjohann

**Affiliations:** † Materials Science Center, 53405 National Renewable Energy Laboratory, Golden, Colorado 80401, United States; ‡ Materials Physics Department, Sandia National Laboratories, Livermore, California 94550, United States

**Keywords:** LaCoO_3_, STEM, EELS, in situ, complex oxides, spin transition

## Abstract

The high-temperature spin and electronic transitions in LaCoO_3_ have recently been leveraged to create neuromorphic (brain-inspired) devices. While these devices have shown the potential for impactful functionality in next-generation computing systems, the nanoscale dynamics of the spin and electronic transitions that underlie their operation are not well understood. Inhomogeneities related to interfaces, electrode contacts, strain, and crystal defects can all affect device performance, making nanoscale characterization of the transitions essential for producing consistent and reliable devices. Here, we demonstrate the first nanoscale in situ measurement of the spin transition in LaCoO_3_ at device-relevant temperatures (25–325 °C) over length scales of tens of nanometers using STEM-EELS. This measurement is enabled by an Al_2_O_3_ coating, which prevents unwanted reduction of the LaCoO_3_ specimen at high temperature and vacuum. The detailed understanding of LaCoO_3_ transition dynamics enabled by such measurements will be crucial for optimizing LaCoO_3_-based neuromorphic devices and increasing reliability for real-world application.

## Graphical abstract

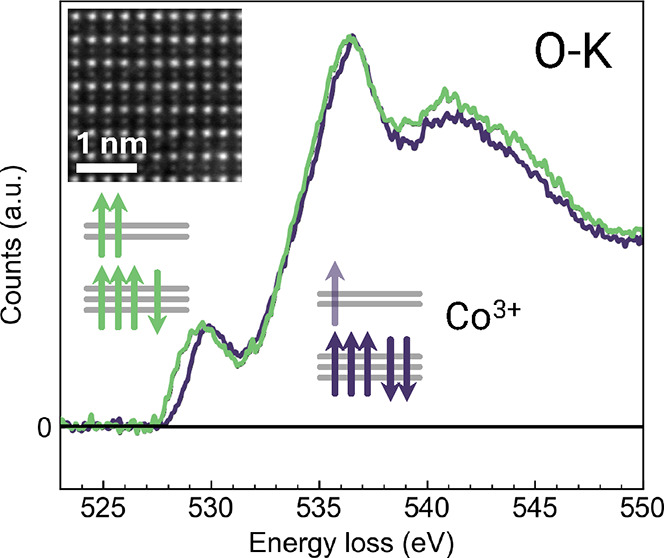

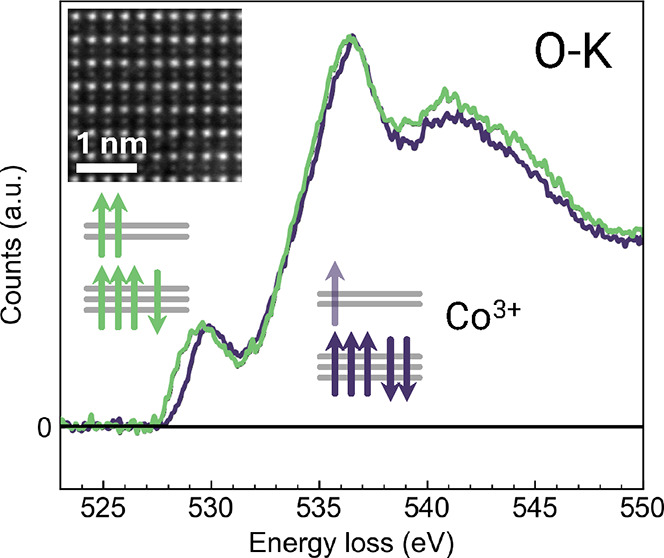

Lanthanum cobalt oxide (LaCoO_3_) has garnered significant research attention over the last several decades due to its strongly correlated nature, which gives rise to intriguing electronic and magnetic properties.^1–4^ While LaCoO_3_ has historically been used in solid oxide fuel cell cathodes^5,6^ and catalysis,^7,8^ interest has grown in its use for advanced microelectronics applications, including brain-inspired (neuromorphic) computing.^9–14^ For example, the broad, above-room-temperature electronic transition exhibited by LaCoO_3_ has recently been leveraged to demonstrate biological axon-like active signal transmission in an adjacent conductor.^12^ The discovery enables electrical signal amplification without breaking the conductor to introduce additional amplifiers in the circuit. Breakthroughs like this justify pursuing the nanoscale integration of materials like LaCoO_3_ into the next development phase of neuromorphic computing devices.

Though LaCoO_3_ has shown promise as a switchable electronic material, the physics underlying the spin and electronic transitions in LaCoO_3_ are still not fully understood, limiting material development and opportunity for implementation in reliable, nanoscale neuromorphic devices. It is accepted that the interplay between spin, electronic, and lattice degrees of freedom in LaCoO_3_ leads to a spin transition at low temperature (∼90 K) and a broad semiconductor-to-metal transition (SMT) above room temperature (∼300–600 K), which is concomitant with a further broad spin transition.^15–17^ This complexity arises from the Co ion, which has a nominal formal charge of 3+ and thus a partially filled 3*d* band. Comparable values for the interatomic exchange energy (*J*_H_) and crystal field splitting (10 Dq) between the *e*_*g*_ and *t*_*2g*_ states in LaCoO_3_ mean that the possible spin states are very close in energy (Figure 1a). The preferred state is thus sensitively dependent on the precise crystal field magnitude, which is a function of Co–O bond length and O–Co–O bond angle.^18,19^

**Figure 1:**
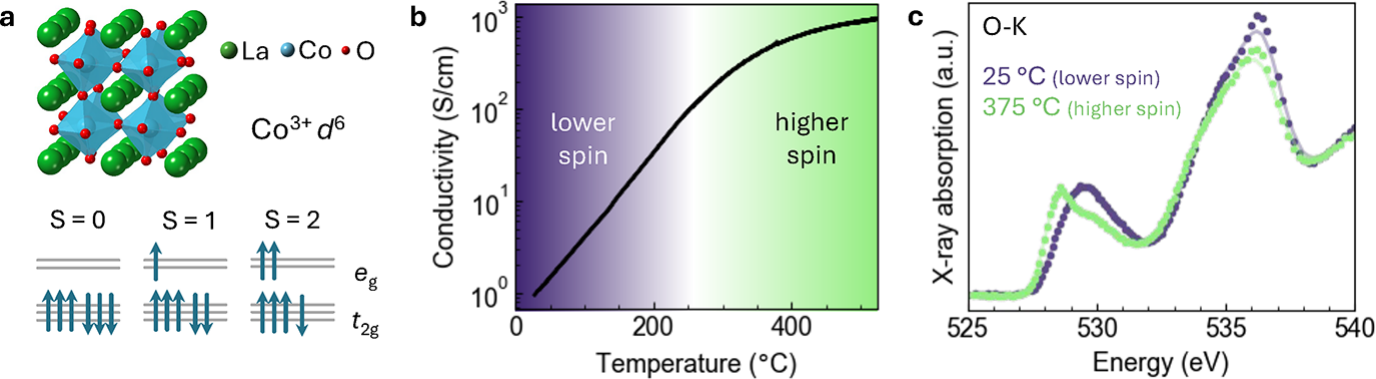
Macroscale structure and electronic properties of LaCoO_3_ thin films. (a) Atomic model of LaCoO_3_ and energy level diagrams for the Co^3+^ ion in the low, intermediate, and high spin states. (b) Temperature-dependent electrical conductivity data showing a broad insulator-to-metal transition between room temperature and 500 °C. (c) X-ray absorption spectroscopy (XAS) O–K edge spectra acquired at room temperature and 375 °C from the same film measured in b, which exhibits a clear shift in the prepeak feature (∼530 eV) toward lower energy at high temperature. The data in c was adapted from ref 13. Available under a CC-BY 4.0 license. Copyright 2024 Woo et al.

In the case of electrothermal oscillator devices used in artificial neurons or axons, which make use of the high temperature SMT and spin transition, the situation is even more complex.^12^ Such nanoscale devices are expected to contain inhomogeneities due to strain, contacting electrodes, and crystal defects, which affect their function. Thus, nanoscale characterization techniques are required to understand the connections among the processing, structure, and ultimate properties of devices. X-ray and neutron scattering methods have been used extensively to study the averaged microscale structural and chemical changes characteristic of the high temperature transitions in LaCoO_3_.^2,4,20,21^ However, the spatial resolutions of these techniques lead to averaging over areas larger than the expected heterogeneities in nanoscale devices, meaning that crucial details could be obscured. When the material is scaled down for such devices, larger-scale probes are unable to capture the effects of interfaces or nanoscopic defects, which can have outsized effects at such small scales. To effectively implement electrical oscillator devices in next-generation computing technology, we need to be able to track and evaluate the spin-state transition in devices at the nanoscale, necessitating nanoscale characterization techniques.

While controversy remains over the exact states associated with the low- and high-temperature transitions, one common interpretation holds that the Co^3+^ ions occupy a mix of three different states (Figure 1a): a low spin state (*t*_2g_^6^, S = 0), an intermediate spin state (*t*_2g_^5^ *e*_g_^1^, S = 1), and a high spin state (*t*_2g_^4^ *e*_g_^2^, S = 2), with the proportion of Co^3+^ ions in each state changing with each observed transition.^3,22^ More complex spin states have also been suggested.^23,24^ Nonetheless, the changing spin states have been reported to produce distinct signatures in the near-edge fine structure of the O–K edge as measured by spectroscopic methods such as X-ray absorption spectroscopy (XAS)^2,13^ and electron energy loss spectroscopy (EELS).^25^

EELS in scanning transmission electron microscopy (STEM) has significant potential for analyzing complex electronic transitions in correlated oxides. It can achieve sub-Angstrom spatial resolution, with energy resolution similar to that attainable by XAS.^26,27^ In situ temperature-controlled STEM experiments enable high-temperature spectroscopic and structural measurements with nano- to atomic-scale spatial resolution.^28,29^ However, the instability of LaCoO_3_ in reducing environments (such as the high vacuum of the STEM sample region)^30^ have so far prevented in situ STEM characterization of the high-temperature transitions in LaCoO_3_. Though the low-temperature spin transition in LaCoO_3_ has been previously measured at the nanoscale by STEM-EELS,^25^ the high temperature transition has not.

Here, we leverage the combined spatial and energy resolution of STEM-EELS to measure the spin-state transition in LaCoO_3_ between room temperature and ∼300 °C over a total field of view of ∼90 nm × 90 nm. The measurement was enabled by a method to conformally coat LaCoO_3_ STEM specimens, creating an effective barrier to oxygen loss during heating under high vacuum. The ability to measure the LaCoO_3_ spin state at the nanoscale will enable the characterization of local heterogeneity in the state transition, including in devices currently under investigation for neuromorphic computing applications. Detailed understanding of transition dynamics will be crucial for developing these devices to be consistent and reliable.

LaCoO_3_ thin films and freestanding LaCoO_3_ flakes were grown by using pulsed laser deposition (PLD) on LaAlO_3_(100) substrates (see Supporting Information for growth details). Raman, X-ray photoelectron spectroscopy, and X-ray Diffraction and Reflectivity data were acquired to determine film quality and thickness (see Supporting Information Figure S1). The LaCoO_3_ thin films exhibit the expected broad SMT as a function of temperature above 25 °C (Figure 1b). As previously reported for these LaCoO_3_ films, XAS O–K edge spectra acquired at room temperature and 375 °C exhibit a clear shift in the prepeak feature at ∼530 eV (Figure 1c).^13^ This result is consistent with earlier studies based on bulk LaCoO_3_, which attribute it to the low-to-high spin-state transition.^2^ While this provides clear evidence of the spin-state transition in these thin films, XAS cannot provide sufficient spatial resolution to investigate the effects of local heterogeneity on the transition, which in turn influences the operation of oscillator devices of interest for neuromorphic applications. These heterogeneities are expected to occur on the scale of Angstroms to hundreds of nanometers, well below the spot size of X-ray sources. STEM-EELS measurements can be performed with down to sub-Angstrom resolution and probe nominally the same electronic transitions as validated by XAS.

LaCoO_3_ TEM samples were prepared on commercial microelectro-mechanical system (MEMS) heating chips in two geometries: focused ion beam (FIB) lamellas (Figure 2a–c) and freestanding flakes (Figure 2d–f) (see Supporting Information for sample preparation and STEM imaging details). Figure 2 presents an overview of these sample geometries as imaged by scanning electron microscopy (SEM) and STEM. The cross-sectional lamella samples exhibit an ∼5 nm epitaxial layer at the substrate interface, above which the film relaxes via the formation of misfit dislocations (Figure 2c). The dislocations are also clearly visible in the plane-view flake samples (Figure 2f), where they appear to form a random network of vertical and horizontal line defects. A few of the line defects are indicated with arrows in the same image reproduced in Supporting Information Figure S2. While the abundance of these dislocations was not found to suppress the spin and insulator-to-metal transitions, they may affect the temperature range and dynamics of these transitions.

**Figure 2:**
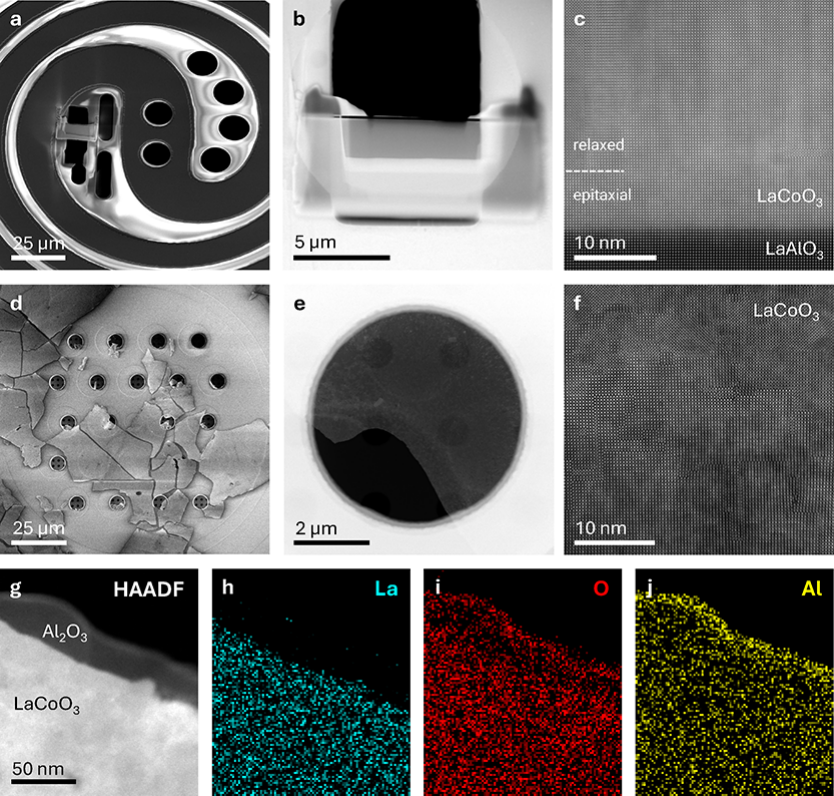
LaCoO_3_ scanning transmission electron microscopy (STEM) sample preparation. (a–c) Scanning electron microscopy (SEM) (a) and high-angle annular dark-field (HAADF)-STEM (b,c) images of a focused ion beam (FIB) lamella placed on an in situ heating chip for a TEM heating holder. Epitaxially strained and relaxed layers of the LaCoO_3_ film are labeled in c. (d–f) SEM (d) and HAADF-STEM (e,f) images of freestanding flakes mechanically transferred to an in situ heating chip for a TEM heating-biasing holder. (g–j) Simultaneously acquired HAADF-STEM image (g) and energy dispersive X-ray spectroscopy (EDS) elemental maps (h–j) for La, O, and Al, respectively, showing a continuous ∼15 nm Al_2_O_3_ coating extending over the edge of a flake.

For STEM in situ heating experiments, prepared LaCoO_3_ samples were coated with a ∼10 nm layer of amorphous Al_2_O_3_ using atomic layer deposition (ALD) to form a conformal layer (Figure 2g, see Supporting Information for deposition details). This coating prevents the loss of oxygen from the LaCoO_3_ during heating due to the reducing environment created by the high-vacuum of the STEM column. At the same time, the low Z number of the constituent elements and the amorphous nature of the film prevent it from contributing significant contrast to the STEM images. The coating thickness was chosen to balance effective barrier properties with deterioration of STEM-EELS data. Ten nm was estimated to be well above a minimum thickness for blocking oxygen loss based on previous studies of oxygen diffusion in amorphous ALD Al_2_O_3_,^31^ and we found it did not noticeably degrade the clarity of the EELS O–K prepeak analyzed herein. The consistency and thickness of the Al_2_O_3_ coating was confirmed using STEM energy dispersive X-ray spectroscopy (EDS) as shown in Figure 2g–j. To the best of our knowledge, this is the first reported use of Al_2_O_3_ as a barrier to sample evolution during S/TEM analysis.

Initially, amorphous carbon was employed as a barrier coating (see Supporting Information Figure S3), as it was previously shown to prevent ion diffusion during STEM imaging of beam-sensitive metal halide perovskite materials.^32^ However, amorphous carbon did not suppress oxygen loss sufficiently for the measurement of the LaCoO_3_ spin-state transition. This is perhaps due to inadequate density of the thermally evaporated carbon. Graphene has also been employed as an encapsulation layer to prevent sample damage during TEM imaging.^33^ This method would be highly challenging in the present case, however, due to the geometry of the LaCoO_3_ samples and the underlying MEMS heating chips. Therefore, ALD coating was pursued, as it is well-understood to produce reliable, thin, and pinhole free coatings.

EELS O–K edge spectra of an Al_2_O_3_-coated LaCoO_3_ flake sample acquired at 25 and 300 °C over tens of nanometer fields of view (Figure 3a) show a distinct shift in the prepeak feature (peak i) toward lower energy at elevated temperature. This prepeak is well-understood to represent hybridization between the O 2*p* and transition metal (Co) 3*d* orbitals,^34^ while peaks ii and iii are commonly attributed to hybridization with the A site (La) 5*d* and transition metal (Co) 4*sp* orbitals, respectively.^2^ Here, a shift in the prepeak (peak i) to lower energy indicates an increase in available lower energy *t*_*2g*_ orbitals, as electron density shifts to the higher energy *e*_*g*_ orbitals due to the spin-state transition. Note that the prepeak feature is not affected by the O–K edge signal from the Al_2_O_3_ coating, as the onset of that signal occurs at ∼533 eV (see Supporting Information Figure S4a). Thus, the alumina only contributes to an intensity increase and broadening of peaks ii and iii from ∼533–545 eV, which are not used in this analysis. Additionally, O–K edge spectra were acquired before and after Al_2_O_3_ coating and their features compared to ensure that the coating process did not cause appreciable reduction of the LaCoO_3_ samples prior to in situ experiments (see Supporting Information Figure S4).

**Figure 3:**
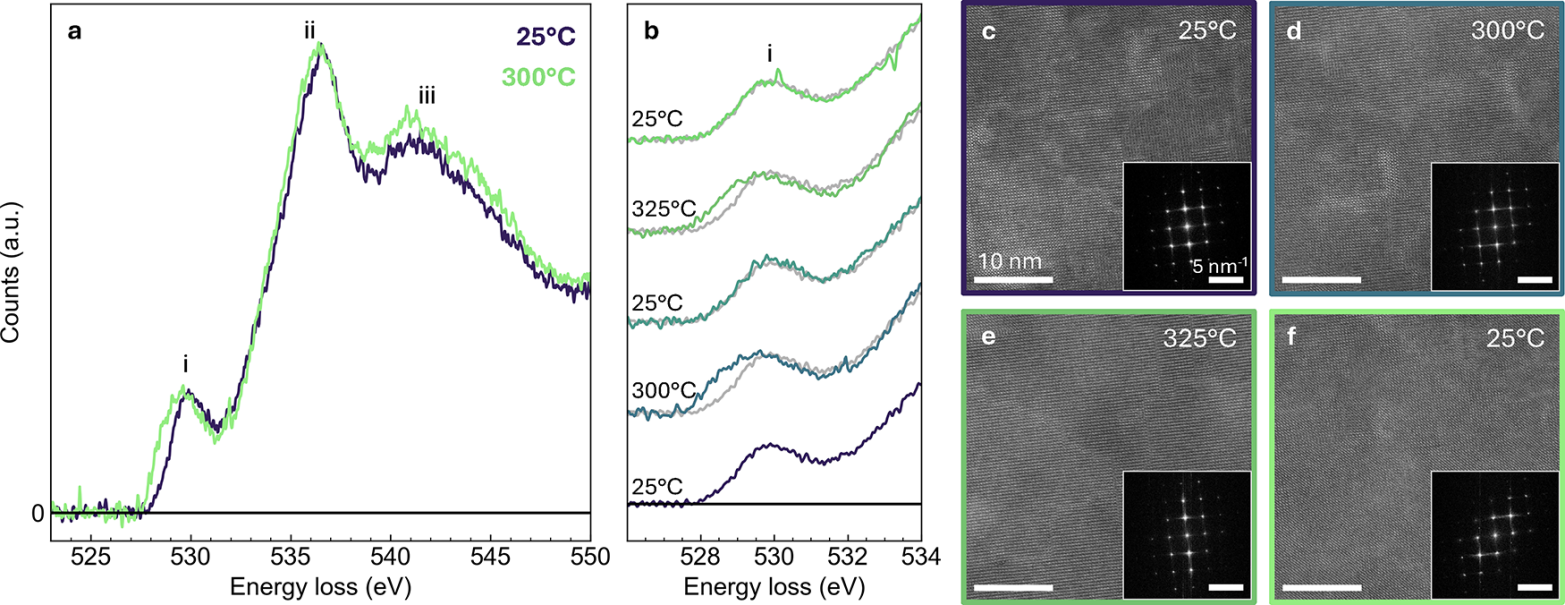
In situ STEM electron energy loss spectroscopy (EELS) measurement of the LaCoO_3_ spin transition. (a) O–K edge EEL spectra of an Al_2_O_3_ coated LaCoO_3_ flake acquired at 25 and 300 °C. The prepeak feature (labeled (i)) exhibits a clear shift to lower energy at elevated temperature, indicating a transition to a higher spin state. (b) Prepeak region of the O–K edge acquired during two heating cycles between 25 and 300–325 °C, showing that the shift in the prepeak feature is reversible and repeatable. The initial 25 °C spectrum is repeated in gray with each subsequent spectrum for ease of comparison. (c–f) HAADF-STEM images acquired during in situ heating at 25, 300, and 325 °C and back to 25 °C, respectively, showing no observable changes in structure. The fast Fourier transform (FFT) of each image is included as an inset.

EELS O–K edge measurements were repeated for two heating cycles between 25 and 300–325 °C (Figure 3b, see Supporting Information Figure S5 for full spectra). During cycling, the shift in the prepeak feature at high-temperature is fully reversible. Each subsequent spectrum acquired at room temperature matches the initial spectrum (repeated in gray for ease of comparison). Additionally, the shift is repeatable, showing the same behavior for the two high-temperature cycles. We can, therefore, be confident that there are no unintended irreversible changes to the sample during heating and that the prepeak shift is due to the reversible spin-state transition. We note that heating the sample further to 375 °C does begin to cause irreversible reduction of the film (see Supporting Information Figure S6), which suppresses the spin transition. We discuss this reduction process in detail below.

HADDF-STEM images (Figure 3c–f) show no observable changes to the atomic structure of the LaCoO_3_ flake. All images show a random distribution of dislocations, which do not notably evolve at the studied temperatures. Furthermore, fast Fourier transforms (FFTs) of the images (Figure 3c–f insets) show no change in crystal symmetry, though there may be slight changes to the extent of tilting in the CoO_6_ octahedra that are not resolvable here. These observations are consistent with previous studies reporting the only structural change accompanying the spin-state transition to be a decrease in the rhombohedral distortion of the LaCoO_3_ unit cell.^35^ While measurement of the oxygen octahedral tilts comprising the rhombohedral distortion of the perovskite unit cell is possible, it would require a very precise visualization of the oxygen anions. Such measurements are extremely challenging, even with the double-tilt TEM holder capability necessary to reach the precise crystalline zone-axis. The lack of structural changes noted here also supports the lack of any unintended reactions, such as reduction, that could occur during the experiment.

Overall, the EELS and structural characterization described herein are consistent with previous X-ray scattering measurements of the high-temperature spin transition.^2,13^ The present measurement technique enables future direct tracking of the spin transition across nanoscale features such as those likely present in electrothermal oscillator devices for neuromorphic computing.^12^ This type of characterization will be critical for optimizing device function and increasing consistency and reliability for real-world implementation.

In situ STEM measurements performed on LaCoO_3_ without an Al_2_O_3_ coating, revealed significant reduction of LaCoO_3_ upon heating (Figure 4). This reduction suppresses the spin transition and thus prevents its measurement. Heating a FIB lamella to just 100 °C and cooling back to room temperature reveals irreversible reduction of the LaCoO_3_ (Figure 4a), as evidenced by the decrease in prepeak (peak i) intensity in the spectrum obtained at 25 °C after cooling as compared to the spectrum acquired prior to any heating (repeated in light gray for ease of comparison). This reduction continues when the temperature is raised incrementally to 200 and then 300 °C, with the prepeak intensity decreasing progressively with the oxygen content.

**Figure 4:**
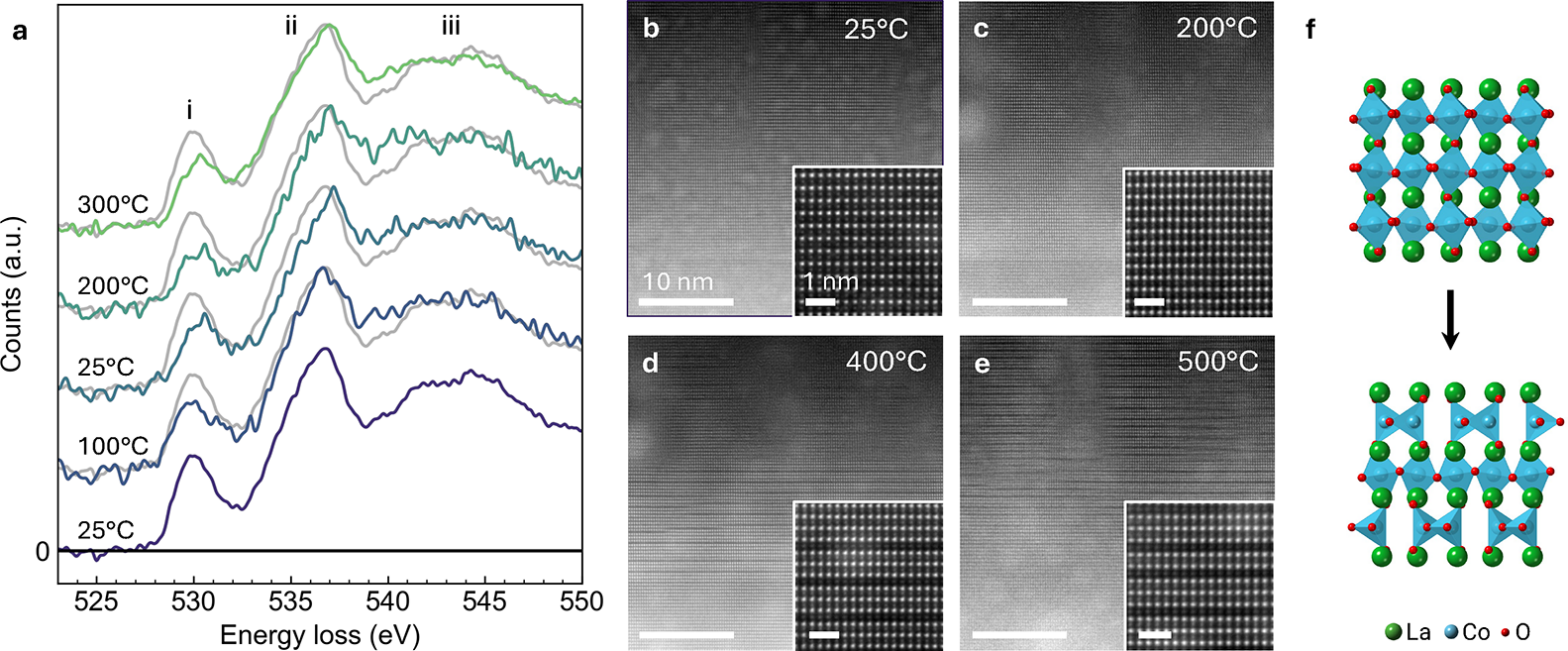
In situ heating of uncoated LaCoO_3_ cross-sectional FIB lamella. (a) O–K edge EEL spectra of the uncoated LaCoO_3_ acquired during two heating cycles, exhibiting irreversible changes to the prepeak feature (labeled (i)) after heating to only 100 °C. The initial 25 °C spectrum is repeated with each subsequent spectrum for ease of comparison. (b–e) HAADF-STEM images acquired during heating at 25, 200, 400, and 500 °C, respectively, showing the formation and increasing frequency of dark horizontal lines, indicative of ordered oxygen vacancies. The insets show a magnified field of view for each temperature. Low-frequency intensity variation is due to residual tungsten from FIB lamella preparation on the sample surface. (f) Atomic models representing the transition from the perovskite (LaCoO_3_) to brownmillerite (LaCoO_2.5_) structure upon loss of oxygen from the sample.

Whereas HAADF-STEM imaging initially shows no change in the atomic structure of the uncoated specimen, it reveals dark lines beginning to form in the LaCoO_3_ lattice when heated to 400 °C (Figure 4b–e). These lines form in CoO_2_ layers starting at random intervals before forming in approximately every third layer and finally every second layer. The dark lines are caused by ordered oxygen vacancies in those layers. Ordering in every other CoO_2_ layer is characteristic of the brownmillerite (LaCoO_2.5_) structure. This transition from the perovskite to an intermediate and then the brownmillerite structure in LaCoO_3‑δ_ (Figure 4f) has previously been observed using electron beam-induced reduction in the STEM^36^ and is consistent with phase behavior in bulk LaCoO_3‑δ_.^37^ To the best of our knowledge, it has not been previously demonstrated by in situ heating. This data also conclusively shows that the dark lines observed in CoO_2_ layers of LaCoO_3_ arise from oxygen vacancy formation, not the spin-state transition, as has previously been suggested.^38,39^

In summary, we have demonstrated in situ measurement of the high-temperature spin-state transition in LaCoO_3_ over length scales of just tens of nanometers. The ability to track the spin transition over these length scales enables characterization of the effects of nanostructure and local heterogeneity on the spin-state transition and concomitant SMT, as well as the dynamics underlying electrical oscillations central to neuromorphic devices like those demonstrated by Brown et al.^12^ These measurements were enabled by a method to prevent reduction of STEM specimens during in situ heating by coating them with a thin, conformal layer of alumina deposited by ALD. This pinhole free, conformal coating ensured oxygen could not escape the specimen during heating in the high vacuum of the STEM column and allowed for EELS measurement of the O–K edge up to 325 °C. The ability to make these measurements with nanoscale spatial resolution and high energy resolution represents an important step toward understanding the structure–property relationships underpinning the function of electrical oscillator devices and therefore optimizing their performance. The coating method developed here also offers a method to protect other sensitive specimens (oxides and otherwise) from reduction during in situ high-temperature STEM characterization.

## Supplementary Material



## Data Availability

The data that support the findings of this study are available from the corresponding author upon reasonable request.

## Notes

Sample growth and STEM experimental details, film characterization, additional STEM imaging and EELS data for carbon- and Al_2_O_3_-coated samples (PDF)
