# Correction to “Nanoscale Tracking of the High-Temperature Spin-State Transition in LaCoO_3_”

**DOI:** 10.1021/acs.nanolett.6c00915

**Published:** 2026-03-06

**Authors:** Michelle A. Smeaton, Elena Salagre, Elliot J. Fuller, Lance M. Wheeler, Katherine L. Jungjohann

In the original publication, the Acknowledgments section is not correct. The following language was incorrectly omitted: “Sandia National Laboratories is a multimission laboratory managed and operated by National Technology and Engineering Solutions of Sandia, LLC., a wholly owned subsidiary of Honeywell International, Inc., for the U.S. Department of Energy’s National Nuclear Security Administration under contract DE-NA-0003525.”

The full Acknowledgments text should read as follows:

“This work was authored, in part, by the National Renewable Energy Laboratory for the U.S. Department of Energy (DOE) under Contract No. DE-AC36-08GO28308. The growth and bulk characterization at Sandia National Laboratories and the sample preparation and STEM characterization of coated samples at NREL were supported as part of the Reconfigurable Materials Inspired by Nonlinear Neuron Dynamics (reMIND) Energy Frontier Research Center funded by the U.S. Department of Energy (DOE), Office of Science, Basic Energy Sciences. STEM characterization of the uncoated sample made use of the electron microscopy facility of the Platform for the Accelerated Realization, Analysis, and Discovery of Interface Materials (PARADIM), which is supported by the National Science Foundation under Cooperative Agreement No. DMR-2039380. Sandia National Laboratories is a multimission laboratory managed and operated by National Technology and Engineering Solutions of Sandia, LLC., a wholly owned subsidiary of Honeywell International, Inc., for the U.S. Department of Energy’s National Nuclear Security Administration under contract DE-NA-0003525. The views expressed in the article do not necessarily represent the views of the DOE or the U.S. Government. The U.S. Government retains and the publisher, by accepting the article for publication, acknowledges that the U.S. Government retains a nonexclusive, paid-up, irrevocable, worldwide license to publish or reproduce the published form of this work, or allow others to do so, for U.S. Government purposes.”

