# Supplementary material for: Nanoscale Tracking of the High-Temperature Spin-State Transition in LaCoO3
Source: Nano Lett. 2025 Nov 5;25(46):16340–5. doi: 10.1021/acs.nanolett.5c03863 (PMC12636071; doi:10.1021/acs.nanolett.5c03863)
Supplement: Supplementary file 1 [file nl5c03863_si_001.pdf]

Supporting Information: Nanoscale Tracking of the High-Temperature Spin-State Transition in $LaCoO_3$

Michelle A. Smeaton,*,† Elena Salagre,‡ Elliot J. Fuller,‡ Lance M. Wheeler,† and Katherine L. Jungjohann*,†

†*National Renewable Energy Laboratory, Golden, CO 80401, United States*

‡*Materials Physics Department, Sandia National Laboratories, Livermore, CA, 94550, United States*

E-mail: michelle.smeaton@nrel.gov; katherine.jungjohann@nrel.gov

Thin film growth and characterization

$LaCoO_3$ thin films of approximately 35 nm were grown using pulsed laser deposition (PLD) on $LaAlO_3$(100) substrates. Freestanding $LaCoO_3$ flakes were grown in the same way but with the addition of a 10-30 nm sacrificial $Sr_4Al_2O_7$ layer between the $LaAlO_3$ substrate and the $LaCoO_3$. $LaCoO_3$ and $Sr_4Al_2O_7$ targets were sourced from Toshima Manufacturing Co, and $LaAlO_3$ substrates were from MTI Co. $Sr_4Al_2O_7$ was grown within an oxygen atmosphere pressure of 10 mTorr at 720 °C, while $LaCoO_3$ was grown at 100 mTorr partial oxygen pressure and 600 °C. All films were grown using a Coherent COMPex 102 excimer laser with wavelength 248 nm and 2.8 J/cm^2 fluence. All samples were annealed in oxygen rich atmosphere after growth to remove remaining oxygen vacancies.

Crystalline quality and phase were assessed using X-ray diffraction and X-ray reflectivity,

Raman, and X-ray photoelectron spectroscopy (XPS) (Fig. S1). XRD indicates the films are relaxed (Fig. S1a), as observed in STEM cross sectional lamellas. The effectiveness of oxygen annealing at removing oxygen vacancies in both thin films on LAO and freestanding films transferred to nitride windows is corroborated by comparison of the vibrational Raman breathing and quadrupole modes (Fig. S1b). Growth of the intended phase and stoichiometry were further supported by XPS measurements (Fig. S1c).

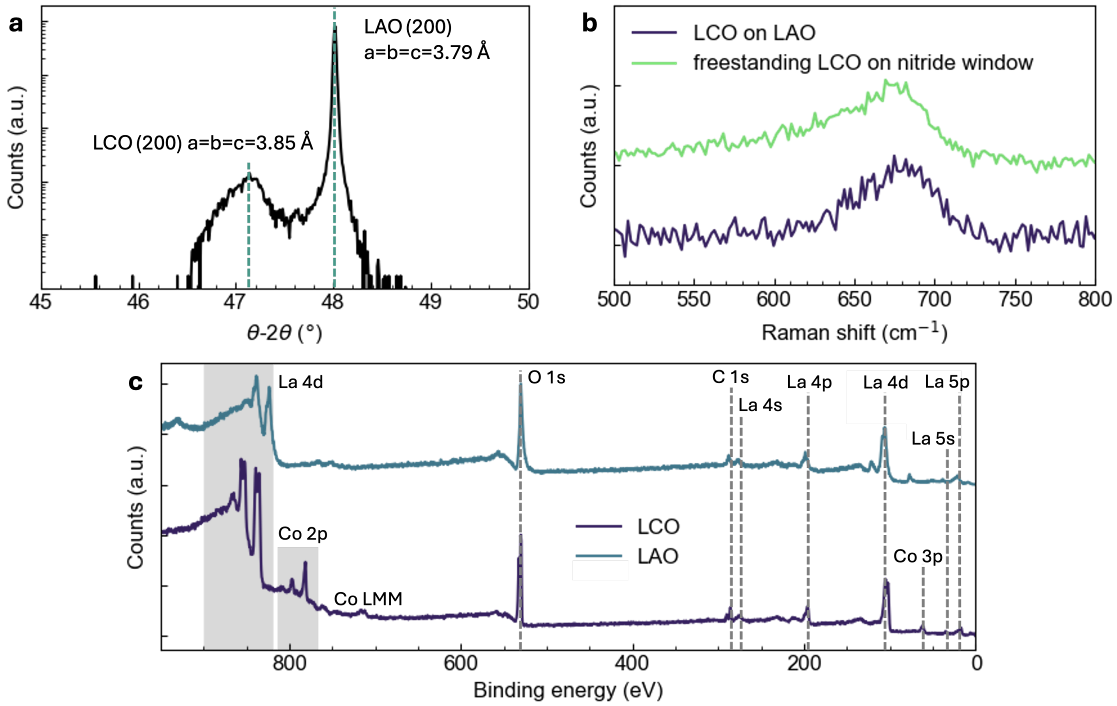

Figure 1: Structural and spectroscopic characterization of $LaCoO_3$ films. (a) XRD of a 55nm thick $LaCoO_3$ film grown on a $LaAlO_3$ substrate showing the (200) diffraction peaks (pseudocubic notation). (b) Raman measurements showing the breathing mode region for an epitaxial $LaCoO_3$ film (purple) and a freestanding $LaCoO_3$ flake deposited on a nitride window (green). (c) XPS measurements of a bare $LaAlO_3$ substrate and a $LaCoO_3$ film (aproximately 60nm thick) on $LaAlO_3$. Although some surface contamination is present (O and C 1s), the stoichiometry is consistent with that of the expected $LaCoO_3$ R-3c perovskite phase.

Temperature-dependent four probe electronic transport measurements were performed from 25 °C to 500 °C in air and compared with reported data (see Fig. 1b in the main text). Electrodes were made of sputtered Pt with a Ta sticking layer. Electrode size was 4 mm x 1 mm with a separation of 0.8 mm. Several dual temperature sweeps were performed at

different speeds to detangle experimental hysteresis from the measurements.

TEM sample preparation

Freestanding films: Post-growth delamination and flake transfer was carried out using the water solubility of $Sr_4Al_2O_7$, as previously demonstrated by Zhang et al.[1] PDMS was deposited on top of the $LaCoO_3$ film, and the stack was submerged in water for a period of 2-6h. After the $Sr_4Al_2O_7$ was dissolved, the PDMS with the attached $LaCoO_3$ film was deposited on top of the desired new substrate (in this case micro-electro-mechanical system (MEMS) TEM heating chips) and warmed on a hotplate to ~50 °C to decrease PDMS-$LaCoO_3$ adhesion. PDMS was then lifted, leaving the freestanding $LaCoO_3$ flakes on the chip.

FIB lamellas: Cross-sectional lamellas were prepared from $LaCoO_3$ films using standard lift out techniques on a Tescan Solaris Ga focused ion beam (FIB). The lamellas were thinned to electron transparency on standard TEM half grids before being transferred to MEMS heating chips and further thinned to ensure minimal surface damage.

ALD coating: TEM samples prepared on heating chips were then coated with ∼ 10 nm of Al_2O_3 using atomic layer deposition (ALD). After loading into the reactor and before deposition, samples were heated to 100 °C and exposed to ozone for 2 minutes to promote oxygenation of the $LaCoO_3$ surface. Depositions were performed at 100 °C with alternating exposures of $Al(CH_3)_3$ (trimethylaluminum [TMA]) and H_2O. The exposed metal contacts on the TEM MEMS heating chips were covered with Kapton tape during the deposition process to avoid affecting the electrical contact and temperature control during in-situ experiments.

STEM imaging and spectroscopy

In-situ scanning transmission electron microscopy (STEM) experiments were performed on multiple S/TEM instruments. STEM imaging and electron energy loss spectroscopy (EELS)

data of Al_2O_3 and carbon coated $LaCoO_3$ samples were collected on a Thermo Fisher Scientific (TFS) Spectra 200 operating at 200 kV with a 24.2 mrad convergence semi-angle and equipped with a TFS Super-X energy dispersive X-ray spectroscopy (EDS) detector and a Gatan Enfinium spectrometer. STEM imaging data of uncoated $LaCoO_3$ samples were acquired using a TFS Titan Themis and a TFS Spectra 300 both operating at 300 kV with a 30 mrad convergence semi-angle. EELS data for these samples were collected using a Gatan Continuum GIF.

EDS elemental maps of Al_2O_3 coated $LaCoO_3$ samples were calculated from net X-ray counts after background subtraction of the peaks. The maps were binned in the spatial dimensions to improve the signal-to-noise ratio.

All EEL spectra presented here were acquired while scanning over ∼30 x 30 nm fields of view with a probe current of 150 pA. Spectra were acquired in dual EELS mode, and the zero-loss peak (ZLP) was used to align the energy loss axis for each spectrum. In most cases, 2-3 individual O-K edge spectra were aligned and subsequently summed to achieve high signal-to-noise spectra sufficient for near-edge structure analysis. To ensure the beam current was low enough that the final spectra were not affected by electron beam damage, several short test spectra were acquired sequentially and compared to ensure there were no changes to the edge structure with acquisition time.

References

(1) Zhang, J. et al. Super-tetragonal $Sr_4Al_2O_7$ as a sacrificial layer for high-integrity freestanding oxide membranes. *Science* **2024**, *383*, 388–394.

Additional Figures

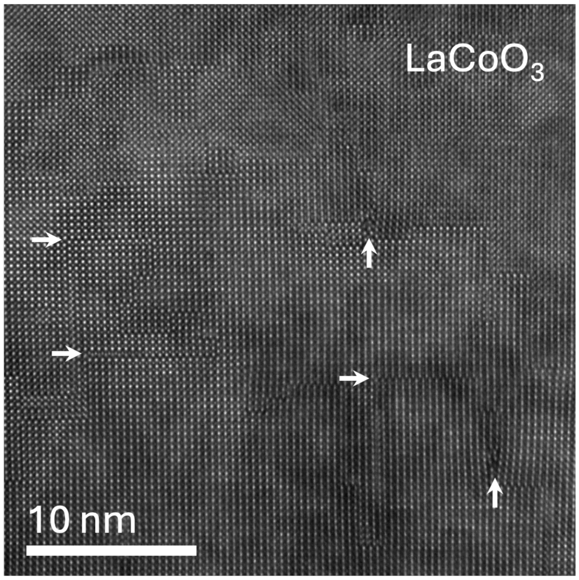

Figure 2: HAADF-STEM image of Al_2O_3-coated $LaCoO_3$ flake sample shown in Fig. 2c in the main text. White arrows indicate a few of the many misfit dislocations present in the flake.

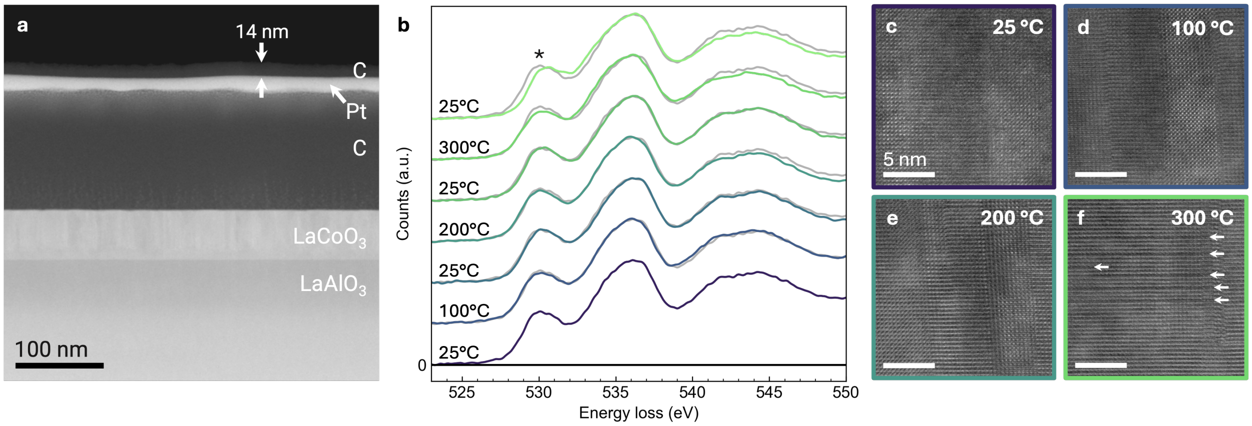

Figure 3: In-situ STEM-EELS of a carbon-coated $LaCoO_3$ lamella. (a) HAADF-STEM image of the sample showing a ∼14 nm thick amorphous carbon layer at the edge of the sample, beyond the platinum and carbon layers deposited during FIB preparation. (b) O-K edge EEL spectra acquired during three consecutive heating cycles. The fine-edge structure remains unchanged when the sample is returned to room temperature after heating to 200 °C. However, after heating to 300 °C, the room temperature spectrum exhibits a suppression of the pre-peak feature (*), indicating irreversible reduction has occurred during heating. (c-f) HAADF-STEM images acquired during in-situ heating at 25, 100, 200, and 300 °C, respectively. At 300 °C, dark lines begin to appear (indicated by white arrows), demonstrating oxygen vacancy formation and ordering.

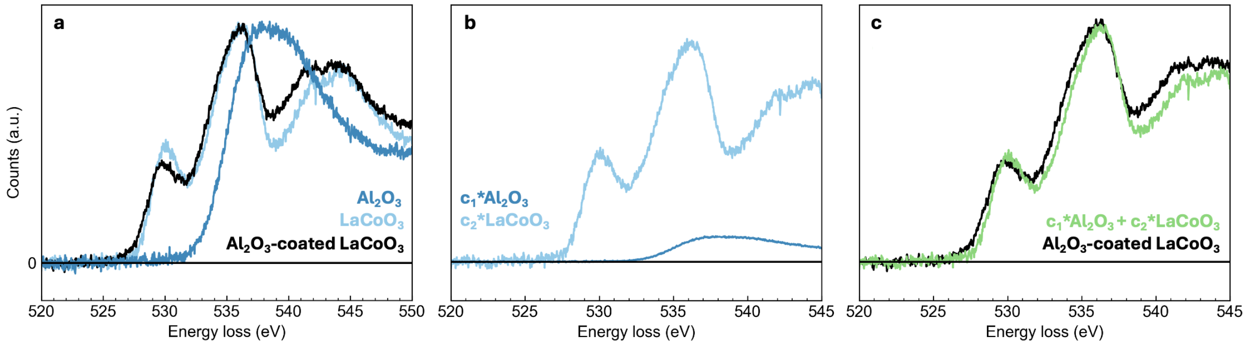

Figure 4: Al_2O_3 coating effects on the O-K edge. (a) Normalized O-K edge EEL spectra of an ALD Al_2O_3 coating and an $LaCoO_3$ sample before and after the coating was deposited. The Al_2O_3 signal does not overlap with the pre-peak feature analyzed in the present study. (b) Al_2O_3 and $LaCoO_3$ O-K spectra weighted according to a least squares fit of the linear combination of spectra $c_1 \cdot Al_2O_3 + c_2 \cdot LaCoO_3$ where $c_1 + c_2 = 1$. The fit was calculated over the energy range 532-545 eV to exclude the pre-peak feature. (c) O-K spectra of the linear combination and the coated $LaCoO_3$ for comparison. The pre-peak feature is slightly higher in the linear combination, indicating that there was slight reduction caused by the coating process. However, the intensity difference (and thus the difference in oxygen vacancy concentration) is very small. We therefore conclude that the Al_2O_3 coating process does not appreciably reduce the $LaCoO_3$ samples.

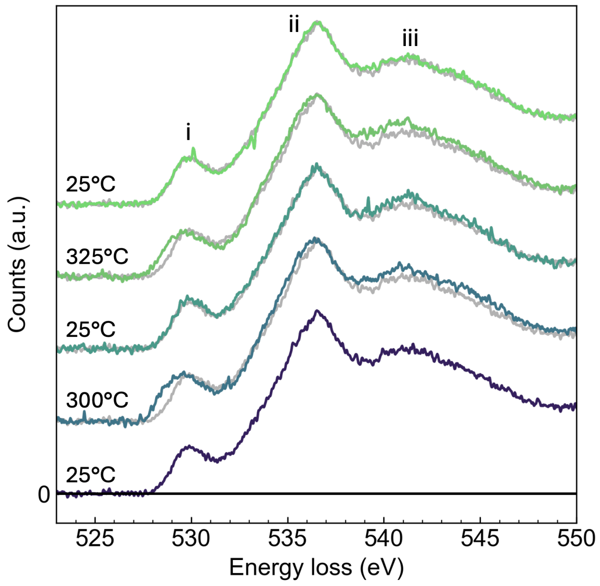

Figure 5: Full energy-range O-K edge EEL spectra of an Al_2O_3 coated $LaCoO_3$ flake acquired during two heating cycles between 25 °C and 300-325 °C, corresponding to Figure 3b in the main text. The initial 25 °C spectrum is repeated with each subsequent spectrum for ease of comparison.

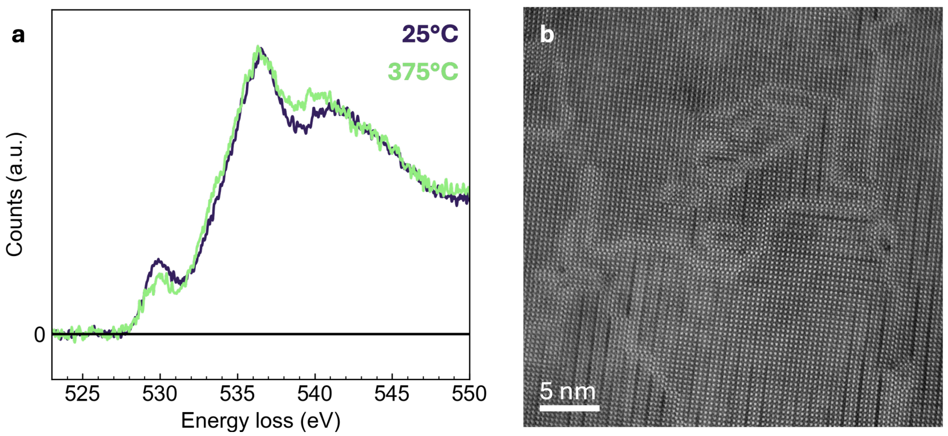

Figure 6: O-K edge EEL spectra of the Al_2O_3 coated $LaCoO_3$ flake acquired 375 °C plotted with the initial spectrum acquired at 25 °C, showing some reduction of the pre-peak feature. (b) HAADF-STEM image of the Al_2O_3 coated $LaCoO_3$ flake after heating to 375 °C, showing dark lines, indicating some reduction and oxygen vacancy ordering has occurred.
